# Molecular and morphoanatomical characterization of *Urocystis heteropogonis sp. nov.*: a novel smut fungus infecting *Heteropogon contortus*

**DOI:** 10.1186/s12870-024-05757-3

**Published:** 2024-11-13

**Authors:** Ayesha Anwar, Hira Wahab, Afshan Wahab, Najam ul Sehar Afshan, Ihab Mohamed Moussa, Khalid M. Elhindi, Meraj Ahmed, Anurag Malik, Mahendra Pratap Singh, Shilpa Gaidhane, Siraj Uddin

**Affiliations:** 1 Department of Botany, University of Peshawar, Peshawar, Khyber Pakhtunkhwa Pakistan; 2 Institute of Botany, University of the Punjab, Lahore, Pakistan; 3 Department of Botany and Microbiology, College of Science, King Saud University, P.O. Box 2455, Riyadh, 11451 Saudi Arabia; 4 Plant Production Department, College of Food & Agriculture Sciences, King Saud University, P.O. Box 2460, Riyadh, 11451 Saudi Arabia; 5 Department of soil science, School of agriculture, Lovely professional University, Jalandhar, India; 6 Division of Research and Innovation, Uttaranchal University, 248007 Dehradun, Uttarakhand India; 7 Center for Global Health Research, Saveetha Institute of Medical and Technical Sciences, Saveetha Medical College and Hospital, Saveetha University, Chennai, India; 8 One Health Centre, Jawaharlal Nehru Medical College, Datta Meghe Institute of Higher Education, Wardha, India

**Keywords:** Poaceae, DNA barcoding, Taxonomy, Basidiomycota, Urocystales, Ustilaginomycetes

## Abstract

**Background:**

A new species of smut fungus, *Urocystis heteropogonis*, was discovered infecting *Heteropogon contortus* in Shawar Valley, Swat district, Khyber Pakhtunkhwa, Pakistan. The study aimed to characterize this fungus based on its morpho-anatomical and molecular features and clarify its phylogenetic position within the genus *Urocystis*.

**Results:**

*Urocystis heteropogonis* was identified as a novel species, distinct from other *Urocystis* species. Morphologically, it is characterized by larger spore balls (14–69 × 11–45 μm) and central spores that are 14–28 × 11–20 μm in size, with each spore containing1–8 central spores. The spore walls measure 0.9–2.5 μm in thickness and the species differs in infection patterns compared to other *Urocystis* species. Phylogenetic analysis based on the ITS and LSU regions of nuclear ribosomal DNA (nrDNA) further confirmed the novelty of the species, placing it within a distinct clade alongside *U. agropyri*, *U. occulta*, *U. piptatheri*, and *U. tritici*.

**Conclusions:**

The discovery of *Urocystis heteropogonis* adds to the diversity of smut fungi infecting grasses and highlights the need for further research into its ecological and agricultural implications. Future studies should focus on the disease’s spread, management, and potential impact on host populations.

## Introduction

Family Poaceae is the fifth largest in terms of species and genera over the world after the Asteraceae, Orchidaceae, Fabaceae, and Rubiaceae families of flowering plants. It comprises 12,000 grass species in 771 genera worldwide, which are divided into 12 subfamilies. The family Poaceae is significant economically as it includes many forage, bamboo and biofuel grass species in addition to Teff (Eragrostis tef (Zucc.) Trotter), wheat (*Triticum aestivum* L.), rice (*Oryza sativa* L.) and corn (*Zea mays* L.) [1]. Depending on the particular microbes and plants involved as well as the current environmental circumstances, plant-microbe interactions can be categorised as advantageous, neutral, or harmful to the plant [2]. By examining these microbes and their potential connections with plants, new and exciting study directions have been opened up.

The genus *Heteropogon*, which belongs to the grass family, was first described in 1807 by Persoon and has two species: *H. glaber* Persoon and *H. hirtus* Persoon. These species are currently categorised as *H. allionii* and *H. contortus*, respectively. There are six species of *Heteropogon* Persoon in the world and they are spread throughout tropical and subtropical regions. *Heteropogon contortus* (Linn.) P. Beauv. ex Roem. & Schult. is the species known to occur in Pakistan, while only India was known to host all six species [3].

*Heteropogon contortus* (L.), a globally distributed member of the Poaceae family, is found in the Sonoran Desert, southern Africa, southern Asia, northern Australia, and Oceania [4–6]. *H. contortus* is known by various names, such as pili grass, black speer grass and tangle head [7–9]. Throughout the world, it is widely used for medicinal purposes. Its extracts possess anti-inflammatory, antibacterial, and antidiuretic properties, used by cultures like the Zulu tribe for treating burns, wounds, and ailments such as toothache, fever, muscle discomfort, anti-inflammatory, hypertension, hematological issues, scorpion sting [6]. Beyond its medicinal uses, *H. contortus* serves as protein-rich fodder for cattle in Australia, Africa, the U.S., and India, and as fiber in India. However, its sharp seeds can harm livestock and pets [7–9].

Smut fungi, classified under the Basidiomycota phylum, are biotrophic pathogens that affect grasses and sedges. They infect the reproductive organs of plants, forming black spore masses known as sori. Favorable conditions for the development of smut fungi include moderate temperatures (25–40 °C) and high humidity. Studying smut fungi is crucial for understanding their life cycle, prevention, and control, particularly due to their detrimental effects on agriculture [10]. With over 1,450 species across 77 genera, smut fungi represent the second-largest group of phytopathogenic fungi after rusts, impacting crops such as sugarcane, wheat, and rice [11].

The genus *Urocystis* Rabenh. ex Fuckel includes over 170 species that primarily form sori on leaves and stems, creating streaks, swellings, and galls. The spore balls contain 1–4 central fertile cells surrounded by colorless sterile cells, with teliospores germinating in situ. Although *Urocystis* is found globally, it is more common in temperate regions [12]. Over 60% of *Urocystis* species are associated with monocotyledons [11–13].

Molecular phylogenetic studies commonly use nuclear ribosomal DNA (nrDNA) regions, such as the Internal Transcribed Spacer (ITS) and Large Subunit (LSU), which are widely applied in fungal taxonomy [14]. However, many recently described *Urocystis* species such as described *Urocystis* species, such as *U. achnatheri* L. Guo, *U. anemones-narcissiflorae* Vánky, *U. arxanensis* L. Guo, *U. beckwithiae* Vánky, *U. circaeasteri* Vánky, *U. dunhuangensis* S.H. He & L. Guo, *U. glabella* Vánky & R. G. Shivas, *U. helanensis* L. Guo, *U. koeleriae* L. Guo, *U. phalaridis* Vánky, *U. puccinelliae* L. Guo & H.C. Zhang, *U. rostrariae* Piątek, *U. sinensis* L. Guo, *U. skirgielloae* Piątek, *U. wangii* L. Guo, and *U. xilinhotensis* L. Guo & H.C. Zhang have been identified solely through traditional morphological methods, without molecular confirmation [15–27]. So far, phylogenetic research has mostly centered on species from graminicolous hosts, particularly those in the Triticeae tribe, leaving the broader relationships within the *Urocystis* genus underexplored. Considering the genus includes over 170 species with a wide geographic distribution, a comprehensive molecular phylogenetic analysis is essential to elucidate their evolutionary relationships, species divergence, host specialization, and taxonomic placement. By combining molecular data with morphological and ecological insights, a more accurate and robust classification system for *Urocystis* can be established, in line with current fungal taxonomy standards.

One hundred and fifteen (115) species of smut fungus have been found in Pakistan. There are roughly twenty-eight (28) species known from Khyber Pakhtunkhwa and ten (10) species from nearby mountain regions in Pakistan [28]. In Shawar Valley of district Swat, *Heteropogon contortus* leaves with smut infections were gathered. This smut can’t be linked to any known species, but microscopic and phylogenetic analyses indicate that it belongs to the genus *Urocystis* Rabenh. ex Fuckel. As a result, it is suggested as a new species here.

## Materials and methods

### Survey of sampling site

**Fig. 1:**
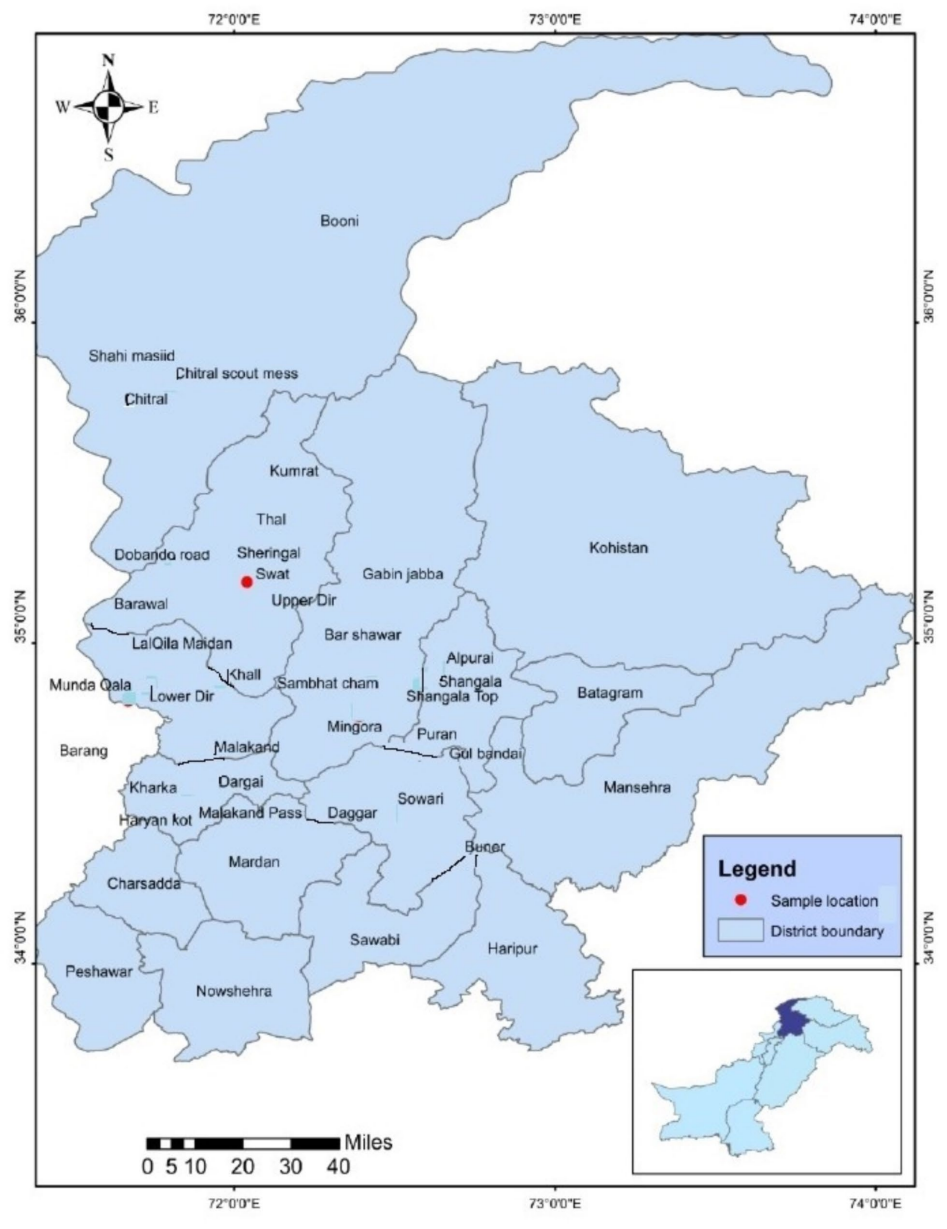
Map of the study area. Legend of the Map: Red Dot: Sample location, Blue Boundary: District boundary within the Malakand division, Map inset: Indicates the location of the region within Pakistan

The infected fungal leaves of *Heteropogon contortus* were taken in April and September of 2020 and 2021 from Shawar valley of Swat district, Pakistan. The Shawar valley is located between longitudes 72°.30’ and 72°.40’ east, and latitudes 34°0.06’ and 34°0.20’ north, placing it in the northeastern direction. The region topography is rough and rocky, with altitudes ranging from 1100 m at Seigram Peak to 3700 m at Cehotasar Peak in the Himalayan region. The total area of valley is 12,192 acres having a population of 20,163 with an overall literacy rate of 16.6% [29]. There is a large range of vegetation in the area. *Quercus oblongata* D. Don and *Quercus dilatata* Royle are two of the dominating deciduous trees. *Pinus wallichiana* A.B. Jacks, *Picea smithiana* (Wall.) Boiss and *Abies pindrow* Royle, are examples of coniferous trees. *Taxus baccata* L. and shrubs are also sporadically found (Fig. 1). The climate and vegetation varies from subtropical to alpine type. The moderate temperature and humidity levels of the region favor the growth of smut fungi, including *Urocystis* species.

### Identification, collection and preservation of plant sample

Formal identification of the *H. contortus* was conducted by Prof. Dr. Sirajuddin, Department of Botany, University of Peshawar, KP, Pakistan, who provided taxonomic verification of the specimen. Smuts were found by first examining the affected parts with a hand lens (10x magnifications) and then a stereomicroscope. The name and date of the sampling area were recorded. After being collected, the plant was shade-dried on blotting paper and then placed in a brown envelope for later use [30] and was given a distinct code number. The sample was brought back to the lab for additional analysis and also deposited at Department of Botany, University of Peshawar, KP, Pakistan with Voucher number PUP Bot. 156 under the following accession numbers OQ565117 (ITS) and OQ565150 (LSU). The mycological survey on fungal diseases of *H. contortus* was conducted in compliance with the guidelines of the Department of Botany, University of Peshawar, Khyber Pakhtunkhwa, Pakistan. Field studies followed the regulations of the Local Government of Swat Forestry Department.

### Microscopic study

Teliospores of smut fungi were carefully removed from the leaves using forceps. For microscopic observations, teliospores were mounted in distilled water and lactic acid, respectively. To prepare permanent slides, a drop of fixing jelly (composed of 60 g gelatin powder, 60 ml glycerol, 2 g copper sulfate, and 100 ml distilled water) was applied to the specimen and secured with a cover slip. The slides were incubated in a refrigerator for 5–10 min for final fixation. A random selection of at least forty (40) teliospores where available, were examined under a LABOMED (CXRII) microscope, and micro-photographs were captured with an HDCE–X5 camera attached to the microscope. Illustrations of teliospores were made using a Lucida camera (Ernst Leitz Wetzlar, Germany). Measurements of at least forty (40) teliospores were taken using ScopeImage 9.0 (X5), noting the smallest and largest values. After data collection, the specimens were described and compared with existing data for accurate species identification.

### Extraction of genomic DNA

For DNA extraction, 200 mg of teliospores of smut fungi was collected from the dried host material with a sterilized needle and placed into a 1.5 ml sterilized centrifuge tube containing three 2-mm glass beads. The tube was then submerged in liquid nitrogen for 1 min, and the spores were homogenized by vortexing at high speed. Following homogenization, DNA extraction was performed using the Gene JET Plant Genomic DNA Purification Mini Kit (#K0791, Thermo Fisher Scientific, Waltham, USA). After grinding, the cracked spores were immediately transferred into a 1.5 ml microcentrifuge tube containing 350 µL of Lysis Buffer A. The mixture was vortexed for 10–20 s to mix thoroughly, followed by the addition of 50 µL of Lysis Buffer B and 20 µL of RNase A. The sample was incubated at 65 °C for 10 min, with occasional vortexing or using a shaking water bath, rocking platform, or thermomixer. Following incubation, 130 µL of Precipitation Solution was added, and the tube was mixed by inverting 2–3 times, then incubated on ice for 5 min. The sample was centrifuged at ≥ 20,000× g (≥ 14,000 rpm) for 5 min to pellet debris. The supernatant (450–550 µL) was carefully collected and transferred into a clean microcentrifuge tube. To this, 400 µL of Plant gDNA Binding Solution and 400 µL of 96% ethanol were added and mixed well. Half of the prepared mixture (600–700 µL) was transferred to a spin column, followed by centrifugation at 6,000 × g (~ 8,000 rpm) for 1 min. The flow-through was discarded, and the remaining mixture was applied to the same column, centrifuging again at the same speed. Next, 500 µL of Wash Buffer I (with ethanol added) was added to the column and centrifuged at 8,000 × g (~ 10,000 rpm) for 1 min. The flow-through was discarded, and the column was placed back into the collection tube. Another wash was performed using 500 µL of Wash Buffer II (with ethanol added), centrifuging at maximum speed (≥ 20,000 × g, ≥ 14,000 rpm) for 3 min. The collection tube was emptied, and the column was re-spun for 1 min at maximum speed to remove any residual ethanol. The spin column was then transferred to a sterile 1.5 ml microcentrifuge tube, and 100 µL of Elution Buffer was added directly to the center of the column membrane. After incubating at room temperature for 5 min, the sample was centrifuged for 1 min at 8,000 × g (~ 10,000 rpm) to elute the genomic DNA. A second elution was performed with another 100 µL of Elution Buffer to maximize DNA recovery. DNA extracts were stored at − 20 °C.

The purified DNA was visualized using ethidium bromide electrophoresis in a 1% agarose gel and viewed under a Gel Documentation System (BIO-RAD Universal Hood II) to confirm its integrity. The nucleic acid was quantified using a NanoDrop 3300 Fluorospectrometer (Thermo Scientific, Wilmington, USA). The purified DNA was then ready for downstream applications or stored at -20 °C.

### Polymerase chain reaction (PCR) and sequencing

Two DNA regions were amplified including the internal transcribed spacers (ITS1-5.8 S-ITS2 = ITS) and large subunit of the nuc rDNA (28 S). 5). The rationale for selecting genes in the phylogenetic analysis of *Urocystis heteropogonis* focuses on using conserved genes, such as rDNA regions (ITS and LSU), which reliably inform evolutionary relationships. The selected genes must be relevant for taxonomic classification; for example, the ITS region is useful for distinguishing closely related species, while LSU aid in resolving higher-level relationships. Previous studies often guide gene selection to facilitate comparisons and build on existing knowledge. The availability of related sequences in databases like GenBank also supports thorough analysis. Overall, these criteria are vital for accurately understanding the evolutionary relationships and classification of *Urocystis heteropogonis*, enhancing insights into its biodiversity and ecological significance. For PCR amplifcation of these two regions, the primer combinations were ITS4/ITS1-F [31, 32] for ITS and LR0R/LR6 [33] for LSU respectively (Table 1). The ITS region was amplified using 34 patterns, each consisting of 30 s at 94 °C, 45 s at 50 °C, and 45 s at 72 °C, followed by a final expansion of 7 min at 72 °C. An underlying denaturation phase took 5 min at 94 °C. Using LROR in conjunction with the reverse complement of LR6, the LSU area was amplified for five minutes at 94 °C as part of an underlying denaturation step. There were thirty patterns of this type, with 45 s at 50 °C, one minute at 72 °C, and a final extension of seven minutes at 72 °C [31–33]. After being separated by electrophoresis on 1% Agarose gel and operated at 110 volts for 50–60 min, the PCR products were stained with GelRed. The PCR products were delivered to TsingKe in China for sequencing after being visualized using the Gel Documentation System default parameters. The sequences generated during this study were submitted to GenBank.

**Table 1:** Primers used in this study

Primer	Region/gene	Sequence 5ʹ to 3ʹ	Reference
**ITS1F (Forward)**	ITS region	CTTGGTCATTTAGAGGAAGTAA	Gardes and Bruns, 1993
**ITS4 (Reverse)**	ITS region	TCCTCCGCTTATTGATATGC	White et al.,1990
**LR0R (Forward)**	LSU region	ACCCGCTGAACTTAAGC	Vilgalys and Hester, 1990
**LR6 (Reverse)**	LSU region	CGCCAGTTCTGCTTACC	Vilgalys and Hester, 1990

### Multiple sequence alignment and phylogenetic analyses

Consensus sequences were created for the forward and reverse primer reads of the newly generated ITS and 28 S sequences using BioEdit v.7.0.9 [34]. BLAST (Nucleotide Blast: https://blast.ncbi.nlm.nih.gov/blast.cg) analysis was used to retrieve highly similar sequences of the nrITS and LSU regions. The maximum query coverage and percent identity of the sequences, along with related taxa, were noted. Sequences retrieved from GenBank and relevant literature were used in an initial alignment, which was trimmed and then realigned using web-PRANK with default settings [35]. For phylogenetic trees, one outgroup sequence was selected. Tamura-Nei model was applied to conduct a maximum likelihood (ML) bootstrap analysis in MEGA6 [36]. Alignment ends were trimmed, ensuring that all sequences had an equal number of sites. The phylogenetic tree reliability was supported by bootstrap values derived from 1000 replicates, with values over 50% indicated on the tree. The newly generated sequences are represented by red dots in the phylogenetic trees (Tables 2 and 3).

**Table 2:** Taxa used in phylogenetic analysis (ITS) with voucher and accession numbers

Taxa	Voucher	ITS	Country
*Urocystis agropyri*	WSP 72,766	KX057795	USA
*Urocystis agropyri*	WSP 72,764	KX057794	USA
*Urocystis agropyri*	WSP 72,769	KX057793	USA
*Urocystis agropyri*	WSP 72,767	KX057784	USA
*Urocystis bolboschoeni*	K:166,455	MN834011	UK
*Urocystis colchici*	RB 2041	DQ875355	Germany
*Urocystis colchici*	GLM-F105784	KY424459	Germany
*Urocystis colchici*	K:181,831	MN834017	UK
*Urocystis eranthidis*	……………………….	HG918043	China
*Urocystis eranthidis*	MR95	KY552912	Iran
*Urocystis eranthidis*	hmk292	JN367299	UK
*Urocystis fischeri*	K(M)188,731	KF668284	UK
*Urocystis fischeri*	K:187,314	MN834015	UK
*Urocystis fischeri*	K:205,043	MN834013	UK
*Urocystis fischeri*	K:205,218	MN834012	UK
*Urocystis heteropogonis*	**PUP Bot.156**	**OQ565117**	**Pakistan**
*Urocystis kmetiana*	1 C	MN855223	Italy
*Urocystis kmetiana*	1B	MN855222	Hungary
*Urocystis magica*	Lluta_2	MK468896	Chile
*Urocystis magica*	Lluta_3	MK468897	Chile
*Urocystis occulta*	WSP 69,497	KX057774	USA
*Urocystis occulta*	WSP 69,121	KX057773	USA
*Urocystis piptatheri*	PUP Bot. 202	MT073413	Pakistan
*Urocystis tritici*	WSP 72,770	KX057780	USA
*Urocystis tritici*	BPI 910,042	KX057781	USA
*Urocystis tritici*	WSP 72,771	KX057779	USA
*Urocystis tritici*	WSP 72,773	KX057782	USA
*Urocystis violae*	1 A	MN855221	Germany
*Phyllactinia angulata*	MUMH928	AB080464	USA

**Table 3:** Taxa used in phylogenetic analysis (combined ITS and LSU) with voucher and accession numbers

Taxon	Voucher	ITS + LSU	Country
*Urocystis agropyri*	WSP 72,766	KX057795	USA
*Urocystis agropyri*	WSP 72,764	KX057794	USA
*Urocystis agropyri*	WSP 72,769	KX057793	USA
*Urocystis agropyri*	WSP 72,767	KX057784	USA
*Urocystis colchici*	K:181,831	MN834017	UK
*Urocystis eranthidis*	MR95	KY552912	Iran
*Urocystis eranthidis*	……………………	HG918043	China
*Urocystis fischeri*	K:205,043	MN834013	UK
*Urocystis fischeri*	K(M)	KF668284	UK
*Urocystis fischeri*	K:205,218	MN834012	UK
*Urocystis fischeri*	K:187,314	MN834015	UK
*Urocystis heteropogonis*	**PUP Bot.156**	**OQ565150**	**Pakistan**
*Urocystis kmetiana*	1B	MN855222	Hungary
*Urocystis magica*	Lluta_2	MK468896	Chile
*Urocystis magica*	Lluta_3	MK468897	Chile
*Urocystis occulta*	WSP 69,497	KX057774	USA
*Urocystis occulta*	WSP 69,121	KX057773	USA
*Urocystis piptatheri*	PUP Bot. 202	MT073414	Pakistan
*Urocystis tritici*	WSP 72,770	KX057780	USA
*Urocystis tritici*	WSP 72,771	KX057779	USA
*Urocystis tritici*	WSP 72,773	KX057782	USA
*Urocystis tritici*	BPI 910,042	KX057781	USA
*Phyllactinia angulata*	MUMH928	AB080464	USA

## Results

### Molecular phylogenetic analysis of nrITS and nrLSU datasets

#### ITS phylogenetic analyses (Fig. 2)

The aligned ITS dataset for phylogenetic analysis comprised 29 nucleotide sequences, which were obtained from both NCBI GenBank and the literature. Our new taxon is shown in red dot in the phylogenetic tree. After the aligned sequences were trimmed at conserved locations at both the 5′ and 3′ ends, there were 964 places in the final dataset. Of these 964 locations, 261 were singletons, 356 were conserved, 350 were variable and parsimony uninformative, and 88 were parsimony informative. Our ITS sequence *U. heteropogonis* sp. nov. (AD81) produced 1019 base pairs of sequence with strong bootstrap support of 98, formed a distinct clade with *U. agropyri* (KX057795, KX057794, KX057793, KX057784), *U. occulta* (KX057773 & KX057774*)*,* U. piptatheri* (MT073413), and *U. tritici* (KX057782, KX057781, KX057780, KX057779). These species showed 92.31–92.92% similarity. *Phyllactinia angulata* (E.S. Salmon) S. Blumer (AB080464) was employed as an outgroup. Our sequence data differs from other species of *Urocystis* available in GenBank. The phylogenetic analysis distinguished it from closer ones. Our taxon is proposed as a new species within the genus *Urocystis.*

### ITS & LSU phylogenetic analyses (Fig. 3)

For the combined ITS and LSU regions, the aligned dataset consisted of Twenty-three (23) nucleotide sequences, which were sourced from both GenBank and the literature. Upon cutting the aligned sequences from both 5′ and 3′ ends, the final dataset consisted of 1001 locations. 401 were variable and parsimony uninformative, 77 were parsimony informative, 331 were singletons and 301 were conserved. A phylogenetic study was performed using *Phyllactinia angulata* (E.S. Salmon) S. Blumer (AB080464) as the outgroup. Our new taxon is shown in red dot in the phylogenetic tree. With a strong bootstrap support of 99, the combined ITS and LSU sequences of our new taxa *Urocystis heteropogonis* sp. nov. (AD81) formed a distinct clade with *U. agropyri* (KX057795, KX057793, KX057784), *U. occulta* (KX057773 & KX057774), *U. piptatheri* (MT073414), and *U. tritici* (KX057782, KX057781, KX057780, KX057779), demonstrating 93.92–94.21% similarity. Thus, the sequences of our taxon do not match the sequence data for other species of *Urocystis*. available in GenBank. So, our taxon is proposed as a new species within the genus *Urocystis*.

**Fig. 2:**
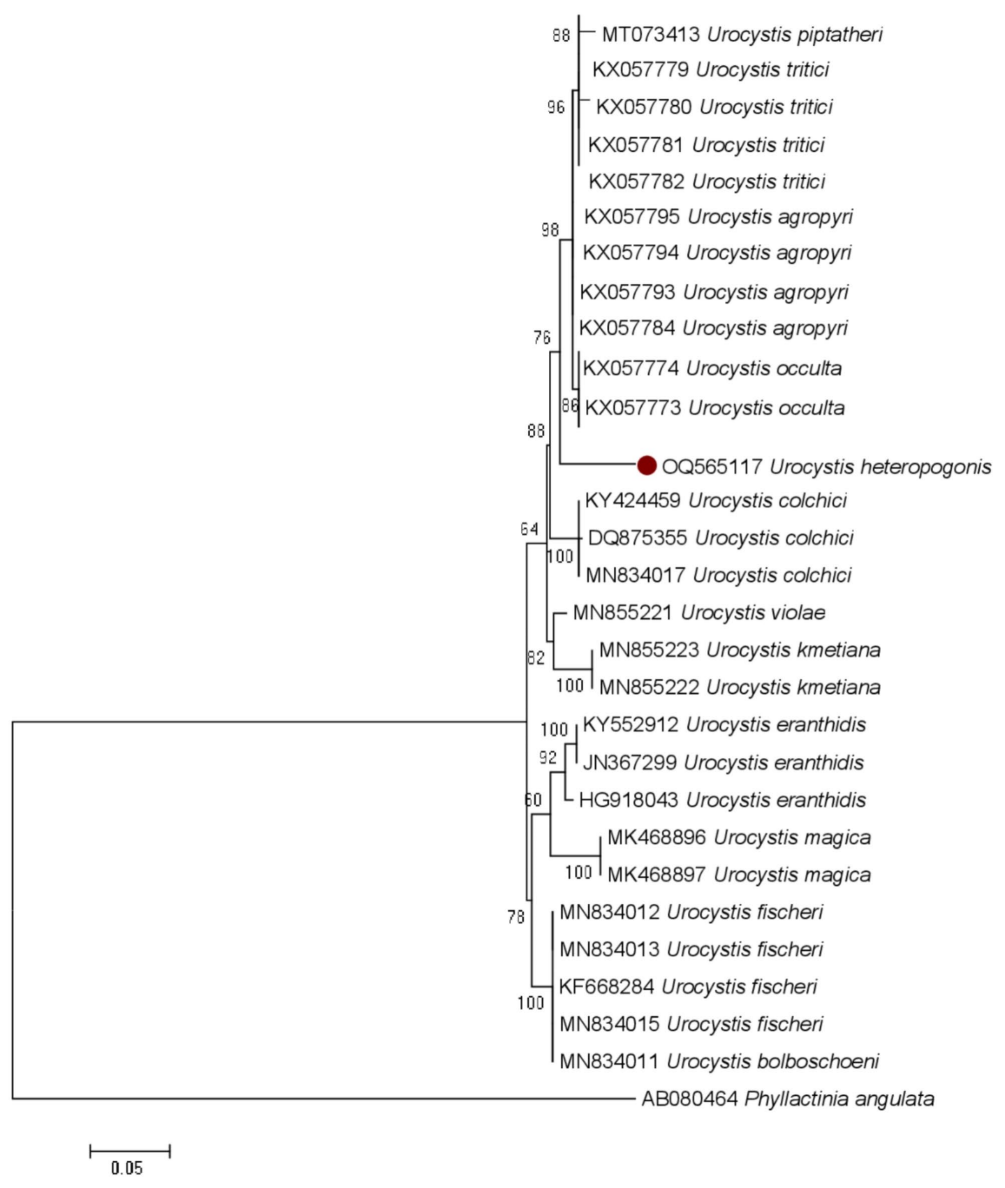
Molecular phylogenetic analysis of ITS sequences of *Urocystis* inferred by using the maximum likelihood method based on the Tamura-Nei model. The tree with the highest log likelihood (-1859.0455) is shown. The percentage of trees in which the associated taxa clustered together is shown next to the branches. The tree is drawn to scale, with branch lengths measured in the number of substitutions per site. Sequences generated from *U. heteropogonis* sp. nov. is shown with red dot

**Fig. 3:**
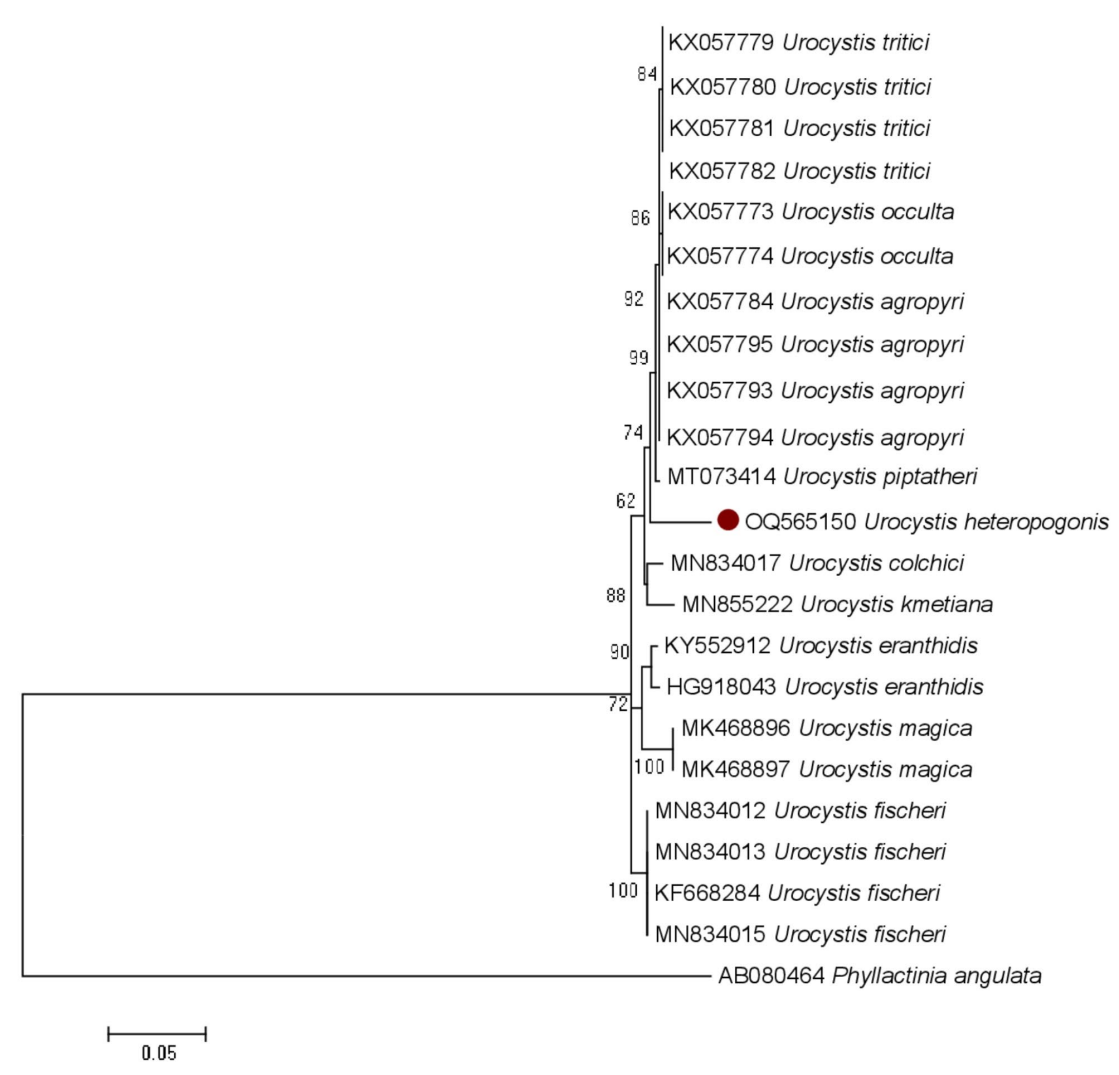
Molecular phylogenetic analysis of combined ITS and LSU sequences of *Urocystis* inferred by using the maximum likelihood method. The tree with the highest log likelihood (-2300.0216) is shown. The percentage in which the associated taxa clustered together is shown next to the branches. The tree is drawn to scale, with branch lengths measured in the number of substitutions per site. Sequences generated from *U. heteropogonis* sp. nov. is shown with red dot

### Taxonomy

#### Urocystis Heteropogonis A. Anwar & Sirajuddin, sp. nov. (Fig. 4)

MycoBank No.: MB855811.

Etymology: —The unique name “*Heteropogonis”* is referred to the host genus *Heteropogon.*

Holotype: **—**PAKISTAN. Khyber Pakhtunkhwa Province: Malakand division, Swat district, Shawar valley, 1200 m.a.s.l, 19 April 2020, Ayesha Anwar (AD81), PUP Bot. 156, GenBank accession numbers OQ565117 (ITS), OQ565150 (LSU).

Diagnosis: **—**Differs from other species of *Urocystis* by size of spore balls, size and number of central spores, spore wall thickness, infection pattern and by the host plant species.

**Fig. 4:**
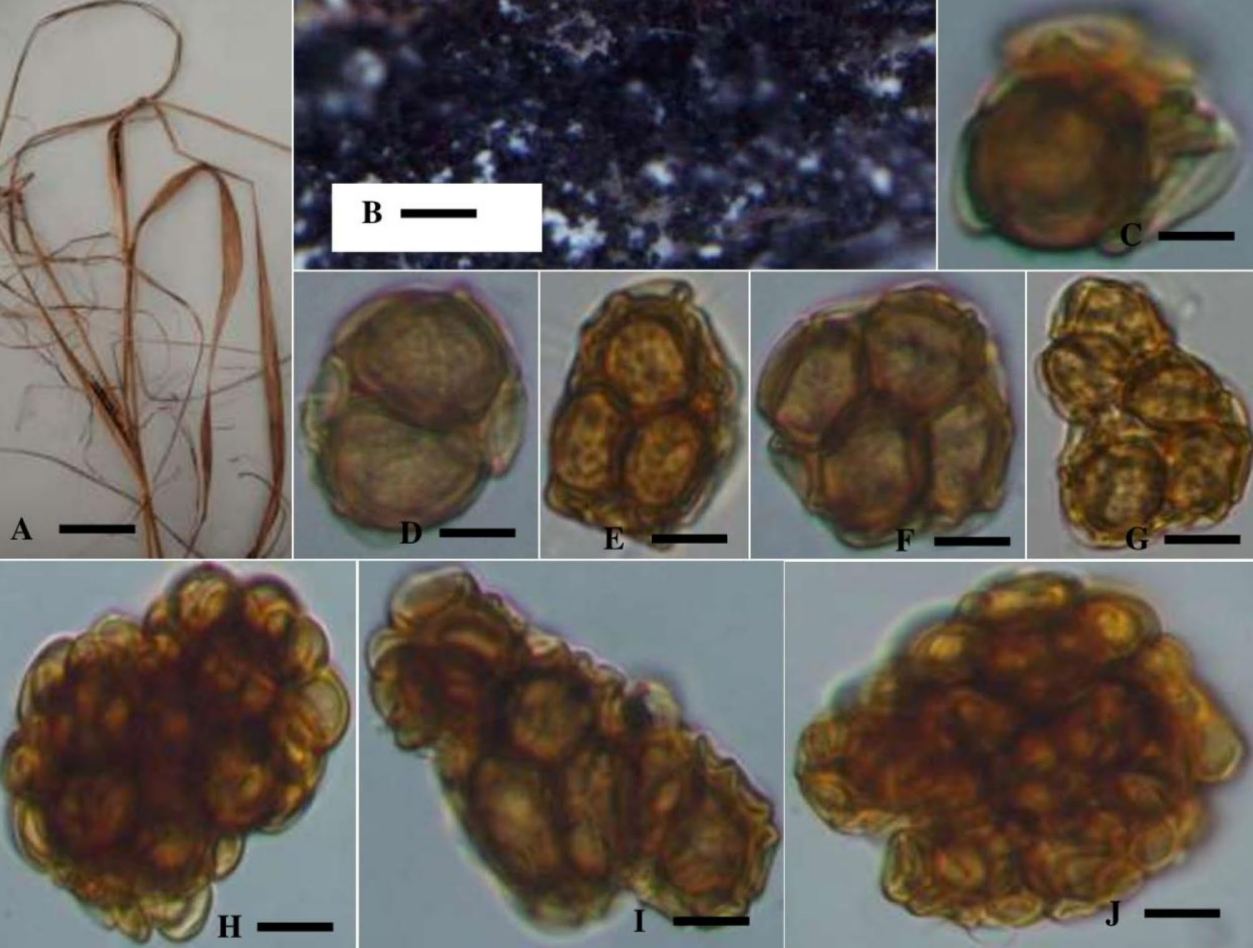
Morphology of *Urocystis Heteropogonis* sp. nov. **A**: Infected host plant *Heteropogon contortus***B**: Stereomicroscopic view. **C**–**J.** Microphotographs of Spore balls. Scale bars: A = 1 cm; B = 2 mm; C = 5.6 μm; D = 5.8 μm; E = 6 μm; F = 4.8 μm; G = 6 μm; H = 8 μm; I = 9 μm; J = 10 μm

Description: —Sori appear as linear striae on leaves that are initially greyish and shielded by the epidermis. When the spores reach maturity, they rupture vertically, revealing a dusty, dark-black mass of spore balls, and at that point, the leaf blade gets twisted. spore balls are globose, subglobose, ellipsoidal, or elongated, 14–69 × 11–45 μm (*n* = 20), light brown to dark reddish brown, wall dark brown, 0.9–1.6 μm thick, composed of 1–8 central spores, frequently fully or partially coated in a layer of sterile cell. Spores are 14– 28 × 11–20 μm in size (*n* = 20), globose, subglobose, ellipsoidal or somewhat irregular, pale yellowish brown or reddish-brown, wall: light brown to dark brown, uniformly thick, 0.9–2.5 μm. Sterile cells are subglobose, smooth, ellipsoidal, ovoid, or rare; they are light yellow, 4–16 × 2–9 μm in size, and have a wall that is 1 μm thick (Fig. 4).

Host and distribution: ––On Poaceae: *Heteropogon contortus*, Shawar, Pakistan. Known only from the type locality.

Additional material examined: —PAKISTAN. Khyber Pakhtunkhwa Province: Swat district, Shawar valley, 1200 m.a.s.l, 15 September 2021, Hira Wahab (HW04), GenBank accession numbers OQ565150 (LSU).

## Discussion

This study introduces *U. heteropogonis* sp. nov. (AD81) as a new species, found on *H. contortus* in Shawar valley, district Swat, Pakistan. Molecular phylogenetic analysis combined with morphological data has confirmed the distinctiveness of this species from other members of the *Urocystis* genus.

*U. heteropogonis* is a newly described species in the Urocystaceae family, distinguished from related species by both morphological and molecular characteristics. Molecularly, phylogenetic analysis of the ITS and LSU regions of nuclear ribosomal DNA places *U. heteropogonis* in a distinct clade, showing 92–94% similarity with closely related species while confirming its classification as a separate species. The combined ITS and LSU analysis further reveals that it forms a unique lineage, clearly separating it from other *Urocystis* species (Figs. 2 and 3). Together, these traits define *U. heteropogonis* as a novel species within the Urocystaceae family. Morphologically, it produces larger spore balls measuring 14–69 × 11–45 μm, containing 1–8 central spores, which sets it apart from other species of *Urocystis.* Additionally, its spore walls are thicker, ranging from 0.9 to 2.5 μm, compared to the thinner walls of related species. The sori form linear striae on the leaves, initially grayish and covered by the epidermis, which later rupture to expose a dark mass of spores. This pattern is unique compared to other *Urocystis* species (Table 4). In comparison, *U. occulta* produces sori on deformed leaves, sheaths, and culms, with smaller spore balls (16–30 μm). *U. tritici* displays sori on leaves and sheaths, with spore balls measuring 20–40 μm and having a distinct reddish-brown wall. *U. agropyri* is found in leaves and culms and has smaller spore balls (16–32 μm) [37], while *U. piptatheri* produces longer striae on leaves and larger spore balls (24–45 μm), though with fewer central spores [38] (Table 4). *U. heteropogonis* was also differs from *U. skirgielloi* M. Piatek, previously identified in India, based on the larger size and composition of its spores [21] (Table 4). Thus, both molecular and morphological evidence strongly support the recognition of *U. heteropogonis* as a new species.

The presence of *U. heteropogonis* has significant ecological impacts on *H. contortus* populations and broader community dynamics. Infected *H. contortus* plants often exhibit stunted growth and reduced reproductive success due to the fungus parasitic nature, leading to fewer viable seeds and diminished plant density. Additionally, *U. heteropogonis* can reduce the abundance of *H. contortus*, allowing other plant species to thrive, thereby altering community composition and affecting herbivore populations that depend on this grass. Field studies have revealed shifts in community structure in grassland ecosystems dominated by *H. contortus*, indicating changes in species richness and biomass linked to infection rates. Overall, *U. heteropogonis* plays a critical role in shaping the fitness of its host and the surrounding ecological community, emphasizing the complex interactions between pathogens and plants in ecosystem dynamics.

*U. heteropogonis* is a newly discovered smut fungus, primarily affects *H. contort* so, comprehensive surveys are needed to accurately map its geographical distribution. To clarify its host range, targeted research efforts are essential, including field surveys to observe its presence on various grasses, experimental inoculation studies to test susceptibility, and molecular techniques to explore genetic relationships with potential host species. Overall, understanding the distribution and potential impact of *U. heteropogonis* on other grasses is critical for enhancing our knowledge of its ecological role.

The discovery of *U. heteropogonis*, has significant implications for conservation efforts involving this grass species and its ecosystems. It highlights the need for biodiversity management by monitoring this pathogen as an indicator of ecosystem health, which can help identify areas under ecological stress. Conservation strategies must protect *H. contortus* by ensuring its health and viability, particularly in areas where it may be declining due to the fungus, potentially through genetic diversity initiatives. The presence of the fungus may also impact essential ecosystem functions, such as soil stabilization and habitat provision, while affecting herbivore populations that rely on this grass. Long-term monitoring of both *U. heteropogonis* and its host is essential for adaptive management, alongside increased funding for research on its lifecycle and impact. Furthermore, raising public awareness about the importance of *H. contortus* and the threats posed by pathogens like *U. heteropogonis* is crucial for effective conservation. Overall, a multifaceted approach is necessary to ensure the health and sustainability of these vital grassland ecosystems.

The discovery and description of *U. heteropogonis* provide a foundational understanding of its pathogenic interactions at both molecular and ecological levels, paving the way for targeted plant health interventions. Insights into the fungus unique morphological and molecular features can inform the development of antifungal agents aimed at disrupting specific biological processes, representing a first step toward therapeutic solutions. Additionally, studying *U. heteropogonis* and its host-specific interactions with *H. contortus* enhances our knowledge of plant immunity, potentially supporting breeding programs to cultivate pathogen-resistant crops. The study also contributes valuable data for integrated pest management (IPM), helping to design biological and ecological control strategies to mitigate the fungus impact. Finally, by identifying specific vulnerabilities of *U. heteropogonis*, the research encourages future exploration of targeted fungicidal compounds and microbial antagonists, advancing environmentally-friendly therapeutic approaches that promote resilient plant ecosystems.

**Table 4:** Comparison of *Urocystis heteropogonis* on the basis of size of spore balls, size and number of central spores, infection pattern and by the host plant species

Species	Host	Sori	Mass of spores	Spore ball (µm)	Spore wall (µm)
***Urocystis heteropogonis***	*Heteropogon contortus*	In leaves	Dusty, Dark-black Powdery	14–69 × 11–45 μm; composed of 1–8 central spores	14– 28 × 11–20; 0.9–2.5 μm
***U. occulta***	*Secale cereale* (cult.)	In deformed leaves, sheaths, culms, spikes	Granular dusty	16–30(–40) × 13.5–20 μm; composed of 1–3(–5) central spores	13–20(–22.5) × 10–13.5 μm; smooth
***U. tritici***	*Triticum aestivum* and *Aegilops* spp.	In leaves, sheaths, stems	Powdery	20–40 × 16–30 μm; composed of 1–3(–5) central spores	12–18(–22) × 10–15 μm; smooth
***U. agropyri***	*Agropyron* spp.,*Elymus* spp.	In leaves, sheaths, culms, often in rachis of aborted inflorescence	Dusty spore	16–32 μm long; composed of 1–3(–5) central spores	12–17.5 × 9.5–15(–16) µm; smooth
***Urocystis skirgielloi***	*Heteropogon contortus*	In leaves	Powdery	composed of 1–4(-7) central spores	12–14 × 9–13 μm; smooth
***Urocystis piptatheri***	*Piptatherum laterale*	In leaves	Powdery	24–45 × 17–36 μm; composed of 1–4(–5) central spores	15–25 × 11–18 μm; 1–2 μm thick

## Conclusion

Our study introduces *U. heteropogonis* sp. nov., a novel smut fungus species isolated from *H. contortus* in Pakistan, supported by both morphological and molecular evidence. This discovery contributes to the understanding of *Urocystis* diversity, revealing unique phylogenetic traits that distinguish *U. heteropogonis* from closely related species within the genus. The findings have broader implications for plant health, as the identified pathogenic interactions provide insights into the impact of smut fungi on host populations and grassland ecosystems. The study also highlights the need for focused conservation and management strategies to protect plant biodiversity, particularly for economically and ecologically important species. Future work should prioritize elucidating the host range, geographic distribution, and potential management approaches to mitigate the impacts of *U. heteropogonis* on *H. contortus* and related species. This research offers a foundation for further investigations into the ecological and agronomic significance of smut fungi, promoting a better understanding of pathogen dynamics in grass ecosystems.

## Data Availability

The data has been uploaded to GenBank and will be accessible before the manuscript is published. The holotype’s accession numbers are OQ565117 (ITS) and OQ565150 (LSU). The corresponding author can provide the data used in this study upon request. The data has been uploaded to GenBank and will be accessible before the manuscript is published. The holotype’s accession numbers are OQ565117 (ITS) and OQ565150 (LSU). The corresponding author can provide the data used in this study upon request.
